# Quantification of 108 illicit drugs and metabolites in bile matrix by LC–MS/MS for the toxicological testing of sudden death cases

**DOI:** 10.1007/s00204-023-03631-z

**Published:** 2023-12-05

**Authors:** Martina Franzin, Rachele Ruoso, Michela Peruch, Gabriele Stocco, Stefano D’Errico, Riccardo Addobbati

**Affiliations:** 1 Institute for Maternal and Child Health, IRCCS “Burlo Garofolo”, Trieste, Italy; 2 Department of Medical, Surgical and Health Sciences, University of Trieste, Trieste, Italy; 3 Azienda Sanitaria Universitaria Giuliano Isontina, Trieste, Italy

**Keywords:** Forensic toxicology, Bile, Postmortem, LC–MS/MS, Quantification

## Abstract

Sudden death could occur after assumption of illicit drugs for recreational purposes in adults or after intoxication in children, and toxicological testing would help identify the cause of the death. Analytical methods sensitive and specific for the quantification of a great number of drugs and metabolites in at least 2 matrices should be used. Bile, collected postmortem, may be considered a specimen alternative to blood and urine to perform toxicological testing because of its extended detection window. The present study proposed a LC–MS/MS method to quantify 108 drugs and metabolites in bile. Compounds belonging to the drugs of abuse classes of amphetamines, benzodiazepines, cocaine derivatives, barbiturates, opioids, z-drugs, and psychedelics were analyzed. The sample preparation is simple and does not require solid-phase extraction. The proposed method showed an appropriate selectivity, specificity, accuracy, and precision of the calibrators and quality controls tested (precision < 15%; accuracy < 100 ± 15%). The sensitivity allowed to identify low amounts of drugs (e.g., morphine limit of detection = 0.2 µg/L; limit of quantification = 1.1 µg/L). There is no significant matrix effect, except for buprenorphine and 11-Nor-9-carboxy-Δ9-tetrahydrocannabinol. Carry-over was not present. Analytes were stable at least for 1 month at − 20 °C. Analyzing 13 postmortem specimens, methadone (50%), and cocaine (37.5%) resulted to be the most prevalent consumed substances; the concentrations quantified in bile resulted to be higher than the ones in blood suggesting bile as a potential new matrix for identifying illicit drugs and their metabolites.

**Supplementary Information:**

The online version contains supplementary material available at 10.1007/s00204-023-03631-z.

## Introduction

Sudden death (SD) could be defined as death occurring in an apparently healthy individual or in one subject whose disease seems not to be so severe to cause a fatal outcome (Lucena 2019). Cardiovascular diseases result to be the most prevalent cause of SD, even if cardiac abnormalities could be not visible during autopsy. Nevertheless, there are cases of non-cardiac SD, sometimes attributable to traumas or to assumption of drugs (Sessa et al. 2021).

In the latter cases, death can occur after assumption of illicit drugs for recreational purposes (usually in young and adults) or after intoxication for errors in assumption (usually in children and older people) (Gaw et al. 2023; Sessa et al. 2021). In detail, the drugs of abuse most commonly associated with overdose mortality include synthetic opioids, psychostimulants, cocaine, benzodiazepines, usually in co-assumption, and heroin (Martins et al. 2015). Furthermore, a recent study documented several cases of fatal poisoning among infants and young children and opioids resulted to be the most common substances attributable to this scenario (Gaw et al. 2023).

In this context, toxicological testing is extremely helpful to forensic pathologists to identify the cause of the death and, together with the clinical information provided by autopsy, will shed light on the forensic case.

Analytical forensic toxicology practices deal with developing and validating analytical methods able to detect a great number of illicit drugs and metabolites derived from them (Ojanperä et al. 2012). In particular, it is customary to first analyze the specimens with screening techniques, such as immunoassay techniques, and then to confirm the identification of drugs of abuse with more specific and sensitive techniques, such as gas chromatography or liquid chromatography coupled with mass spectrometry (GC–MS and LC–MS, respectively) (Drummer 2007).

Also, postmortem toxicology requests the evaluation of the presence and the concentration of illicit drugs on 2 matrices, due to the intrinsic variability of biological material, often subjected to putrefaction (Ojanperä et al. 2012).

Traditional matrices, that toxicology laboratories handle, consist in blood and urine: the former provides information regarding the toxicity and the potential lethal concentration at the time of the death; the latter is an identifier of past drug exposure (Bierly and Labay 2018). Unfortunately, these specimens are not always available for removal and collection (Bierly and Labay 2018).

Bile may be considered an alternative specimen for performing toxicological testing in postmortem cases, especially when traditional matrices are not available (Bierly and Labay 2018; Ferner and Aronson 2018). Bile is easy to collect, large in volume, and the great advantage of this matrix consists in the extended detection window (Bierly and Labay 2018).

Drugs, as well as the illicit ones, are also excreted via the biliary route, mostly if they are lipophilic and with high molecular weight (Ghibellini et al. 2006). Generally, after hepatic metabolism, xenobiotics are excreted into bile canaliculi and transported to the gut, where they can be excreted or reabsorbed (Bardal et al. 2011).

To date, both studies on animals and on postmortem cases shed light on biliary excretion of drugs of abuse, even if they are sometimes dated and performed with different techniques (Ferner and Aronson 2018; Misra et al. 1977). Amphetamines were excreted via biliary route by 16% and, after administration of methamphetamine, the main products in bile within 24 h are methamphetamine and amphetamine, followed by p-hydroxymethamphetamine (Kuwayama et al. 2008). Benzodiazepines are known to be excreted after hydroxylation and conjugation (Tominaga et al. 2016), even if the percentage of elimination via biliary route differs greatly between animal models (Bertagni et al. 1972). Cocaine and its metabolite benzoylecgonine (BEG) were found in higher amount in bile after acute and chronic administration of the drug (Misra et al. 1977). Natural and synthetic opioids, in their conjugated form, are known to be accumulated in bile; for instance, 6-monoacetylmorphine (6-MAM) was fond to be present after assumption of heroin(Al-Asmari 2020), as well as 2-ethylidene-1,5-dimethyl-3,3-diphenylpyrrolidine (EDDP) after methadone (Baselt and Casarett 1972).

As previously mentioned, forensic toxicology requires analytical methods able to detect a great number of illicit drugs and metabolites. To date, to the authors’ knowledge, there is no analytical method able to quantify the great number of drugs and metabolites of the classes mentioned before in bile. Therefore, the present study proposed a LC–MS/MS method to confirm the identification of 108 drugs of abuse and metabolites in bile and compared the concentrations found in bile with the traditional biological matrices. In particular, complete validation data and chromatographic resolution were only shown for 12 compounds, belonging to amphetamines, benzodiazepines, cocaine and metabolites, barbiturates, and opioids, to test the method applicability to each of these illicit drug classes and since these substances are involved in the most frequent intoxication and overdose cases.

## Materials and methods

### Chemicals and reagents

MassTox Drugs of Abuse testing Mobile phases A, B and rinsing solution, MassTox Drugs of Abuse Analytical column, 6Plus1 Multilevel Urine Calibrator SET, MassCheck Drugs of Abuse testing urine, MassTox Drugs of Abuse testing Internal Standard (consisting in deuterated compounds for each analyte and reported in S1_validation), MassTox Drugs of Abuse testing Enzyme solution set, MassTox Drugs of Abuse testing Precipitation reagent, and Dilution buffer were purchased from Chromsystems Instruments & Chemicals GmbH (Munich, Germany).

### Sample collection

Femoral venous blood, urine, and bile were collected postmortem during autopsy by forensic pathologists of University of Trieste and School of Forensic Medicine. Samples of bile free of drugs of abuse was also collected. Specimens for toxicological investigation were carried to the Advanced Translational Diagnostic Laboratory and there stored at − 20 °C until the analysis. All samples used in this study were leftover from routine analyses.

### Calibrators and quality controls

Calibrators (CAL) and quality controls (QC) consist in lyophilized analytes mixture (concentration expressed in µg/L) and were reconstituted with the matrix of interest (water, pure bile free drug and bile diluted 1:10/1:100 in phosphate buffered saline (PBS) (Sigma-Aldrich, Milan, Italy) depending on experimental procedure for validation. Concentrations of CAL and QC for each analyte were reported in S1_validation.

### Sample preparation

Ten microliters of internal standard mix (IS) and 40 µL of β-glucuronidase enzyme, whose efficiency of enzymatic hydrolysis was previously tested by Chromsystems Instruments & Chemicals GmbH (Munich, Germany), were added to 50 µL of sample/CAL/QC. After mixing briefly, samples were incubated for 2 h at 45 °C to allow enzymatic deconjugation. At the end of the incubation, 100 µL of precipitant reagent was added and, after vortexing, the sample was centrifuged for 5 min at 14,500 rpm. To 100 µL of supernatant, 150 µL of dilution buffer was added and 10 µL was injected in the instrument.

The sample preparation for the quantification of drugs of abuse in urine and in blood was performed using MassTox^®^ Drugs of Abuse Testing kit according to the manufacturer instructions.

All the prepared and analyzed samples were resulted positive to screening tests by immunoassays.

### Instrumentation and analytical parameters

Analyses were performed with a HPLC Exion LC 2.0 (Sciex, Milano, Italy) combined with a QTRAP 6500 + system (Sciex, Milano, Italy).

To achieve chromatographic separation, a flow rate of 0.4 mL/min was maintained and the analytes were eluted using the following program: 0–0.2 min isocratic 0% B, 0.2–10.2 linear gradient 100% B, 10.2–12.0 isocratic 100% B, 12.0–12.1 linear gradient 0% B, and 12.1–14 isocratic 0% B. The column oven was set at 30 °C. The injection volume was 10 µL.

Samples were introduced to the mass spectrometer and ionized, positively or negatively depending on the molecule, via electrospray ionization using the following conditions: curtain gas, 40 psig; collision gas, high; ion spray voltage, 4500 V for positive mode and − 4500 V for negative mode; capillary temperature, 450 (°C); and ion source gas, 60 psig.

The list of analytes and compound-dependent parameters, comprising *m/z* ratio of precursor ion and product ions (quantifier and qualifier), entrance potential (EP), declustering potential (DP), collision energy (CE), and collision cell exit potential (CXP) for each analyte divided on the basis of the ionization mode, were reported in S1_validation. Multiple reaction monitoring (MRM) mode was adopted.

### Validation of analytical method and forensic applicability

Analytical method validation was performed according to the most recent International Council for Harmonization (ICH) guidelines (ICH guideline M10 on bioanalytical method validation and ICH guideline Q2(R2) on validation of analytical procedures). In particular, selectivity and specificity were evaluated testing the signals of 6 blank samples, derived from forensic cases without any indication of drug abuse or poisoning, and checking in the chromatograms interfering compounds, respectively. Linearity was assessed constructing calibration curves in 3 different analytical runs and plotting the areas of each analyte normalized on the IS of reference. Determination of the limit of detection (LOD) and the lower limit of quantification (LOQ) was done diluting the lowest concentrated calibrator and evaluating the signal at the retention time of analytes in comparison with the background noise and the accuracy, respectively. In particular, the lowest concentration, whose response was considered detectable, was defined the LOD; instead the lowest concentration, which resulted accurate, was the LOQ. Accuracy and precision were assessed testing 3 levels of QC intra-daily (3 times) and inter-daily (in 3 different runs). Furthermore, matrix effect (ME), recovery (RE), and process efficiency (PE) were calculated according to Matuszewski method (Matuszewski et al. 2003). Stability was evaluated testing QC after 1 week and 1 month of storage at − 20 °C. In order to test the applicability to real samples and to find correlation between the different matrices, postmortem specimens derived from 13 individuals obtained from forensic cases with suspected cause of death due to intoxication or overdose were analyzed.

### Data processing and statistical analysis

Data processing and analysis were performed using Analyst (version 1.7) and Multiquant (version 3.0.2) software. Concentration was calculated normalizing the response ratio of analytes on the one of IS; indication regarding the IS used for the normalization of each analyte was indicated in S1_validation. Calibration curves were fit by linear regression with weighting by 1/*χ*^2^, without forcing the line through the origin. Furthermore, bile:blood and bile:urine ratios were calculated from the mean of the concentrations of illicit drugs of the several forensic cases.

## Results

### Method development

#### Sample preparation

Pure sample of bile or bile diluted (1:10 and 1:100) in PBS were tested to choose the best dilution to work with. Sample dilution 1:100 was chosen for sample preparation because it allowed at the same time lower matrix effect, comparing to dilution 1:10, and quantification of low concentration of analytes.

#### Chromatography

**Fig. 1:**
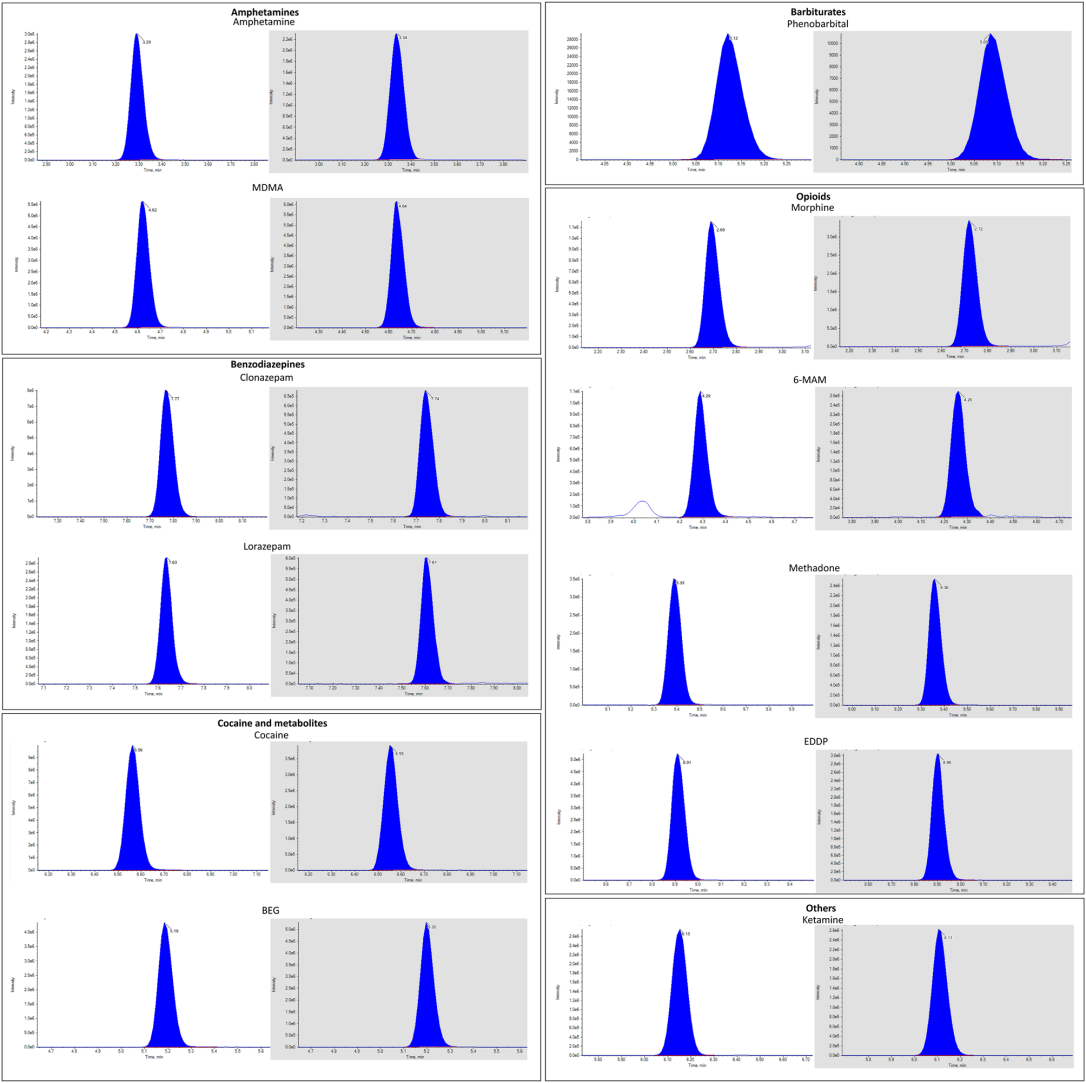
Chromatograms of representative substances belonging to the classes of amphetamines, benzodiazepines, barbiturates, opioids, and other (blank window) and chromatograms of the corresponding IS (grey window)

 The chromatographic conditions allowed separation of all the analytes of interest belonging to the classes of amphetamines, benzodiazepines, booster drugs, cocaine and metabolites, cannabinoids, opioids, z-drugs, barbiturates, and others. The list of all the analytes divided by pharmacological classes and their retention times were reported in S1_validation. Examples of chromatograms of representative substances with the corresponding IS are shown in Fig. 1.

### Method validation

#### Selectivity and specificity

**Fig. 2:**
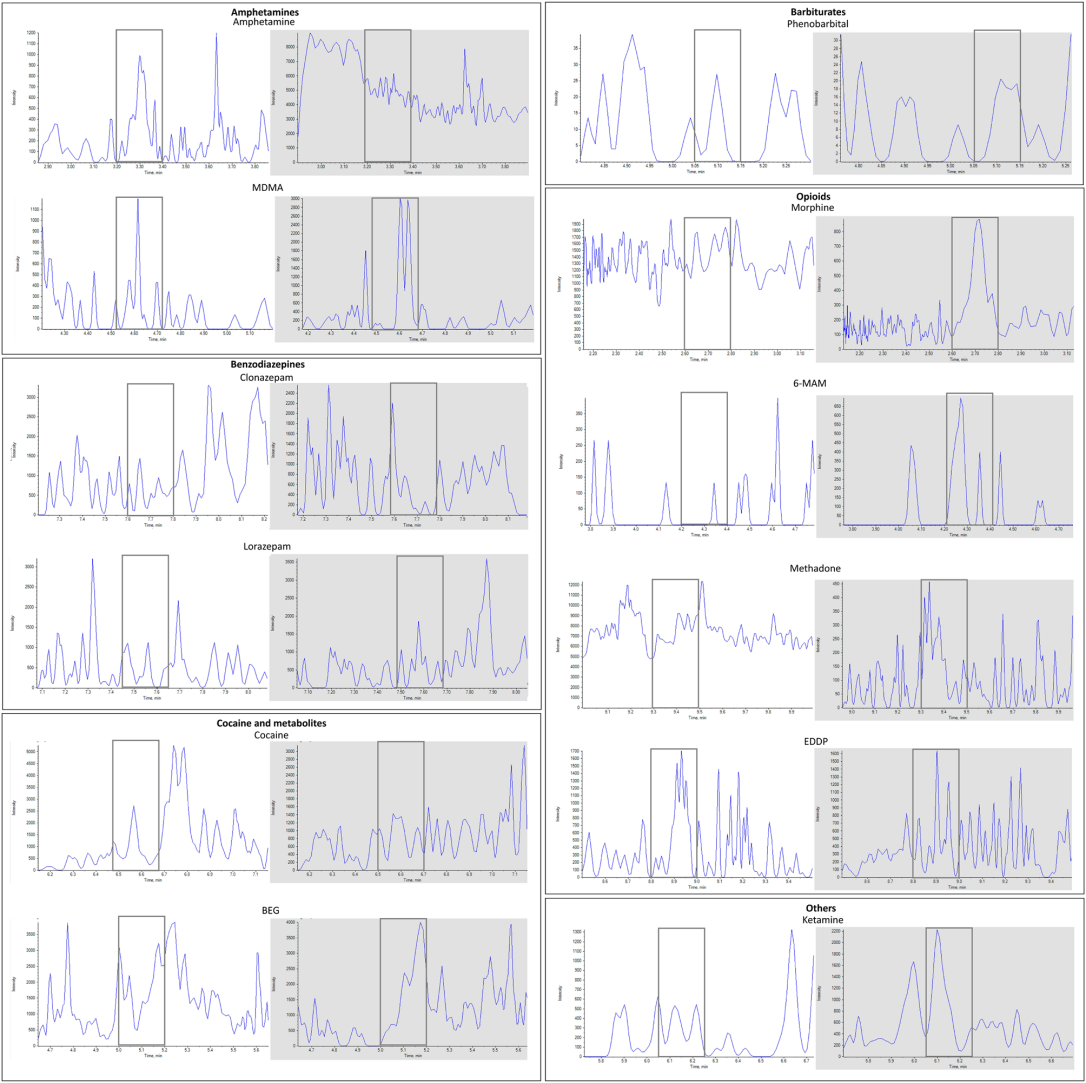
Chromatograms of blanks at the retention times of representative substances belonging to the classes of amphetamines, benzodiazepines, barbiturates, opioids, and other (blank window) and of the corresponding IS (grey window). Regions where analytes may be eluted were marked with grey rectangles

Selectivity was investigated testing 6 blank samples of bile free drug; blanks’ signals were not more than 20% of those of the analytes tested at the CAL with the lowest concentration and than 5% of that of IS at the corresponding retention times (Fig. 2). No interfering compounds different from analytes and IS were noticed.

#### Linearity

**Fig. 3:**
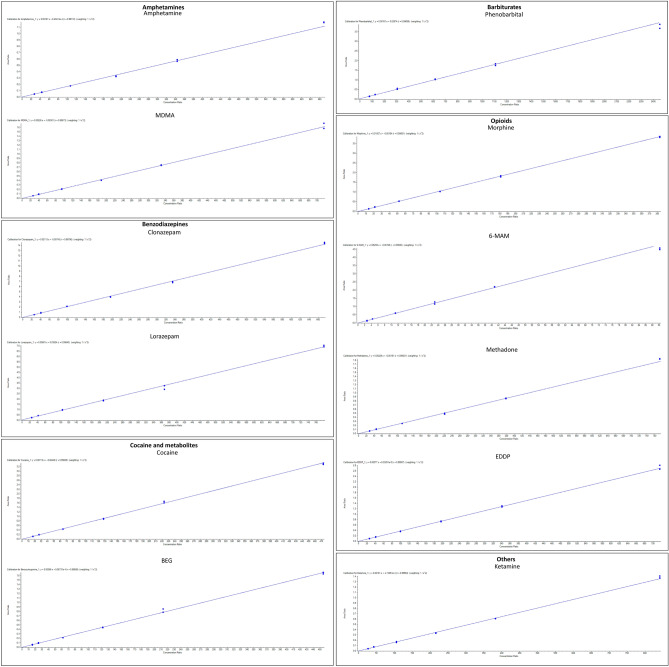
Calibration curves of representative substances belonging to the classes of amphetamines, benzodiazepines, barbiturates, opioids, and other with the indication of the equation of the curve,* R*^2^, and weighting

 Some examples of the calibration curves of representative substances belonging to the classes of amphetamines, benzodiazepines, barbiturates, opioids, and other are shown in Fig. 3. In particular, all curves presented *R*^2^ > 0.99. Furthermore, the intra-day and inter-day precision, expressed as coefficient of variation (CV (%)), was not > 15%. Instead, the accuracy of CAL, expressed as percentages of accuracy (ACC (%)), did not exceed the values of 100 ± 15% of the nominal value after testing intra-daily and inter-daily. Table 1 shows the values of intra-day and inter-day CV (%) and ACC (%) for representative substances of the drugs of abuse classes.

**Table 1:** Intra-day and inter-day precision, expressed as coefficient of variation (CV (%)), and accuracy, expressed as percentage of accuracy (ACC (%)) of CAL

Standard	Nominal concentration (µg/L)	Intra-day						Inter-day	
		I		II		III			
		CV (%)	ACC (%)	CV (%)	ACC (%)	CV (%)	ACC (%)	CV (%)	ACC (%)
Amphetamine									
CAL1	26.90	3.90	99.40	1.40	101.70	0.70	98.50	2.00	99.87
CAL2	43.40	7.50	103.30	1.90	98.40	1.00	103.60	3.47	101.77
CAL3	107.00	0.70	97.30	3.80	98.10	0.60	99.60	1.70	98.33
CAL4	208.00	1.90	94.10	0.60	96.50	0.60	93.40	1.03	94.67
CAL5	344.00	3.40	100.60	4.10	99.80	1.40	101.30	2.97	100.57
CAL6	669.00	0.60	105.60	2.60	105.50	0.10	103.70	1.10	104.93
MDMA									
CAL1	25.80	0.70	98.20	3.80	99.40	5.90	98.50	3.47	98.70
CAL2	39.60	4.40	105.20	2.90	100.80	2.80	100.40	3.37	102.13
CAL3	94.10	2.70	98.70	0.70	99.80	0.10	105.10	1.17	101.20
CAL4	188.00	0.60	96.80	0.90	101.30	4.00	100.40	1.83	99.50
CAL5	330.00	1.20	100.80	5.30	100.40	1.40	98.50	2.63	99.90
CAL6	716.00	5.10	101.20	1.30	98.40	0.90	97.10	2.43	98.90
Clonazepam									
CAL1	25.60	2.40	97.90	3.20	99.80	5.20	101.70	3.60	99.80
CAL2	40.40	9.40	104.60	2.60	99.20	4.80	98.70	5.60	100.83
CAL3	98.80	1.00	103.20	4.30	104.80	4.10	96.60	3.13	101.53
CAL4	196.00	1.80	96.10	2.90	95.40	3.80	99.80	2.83	97.10
CAL5	335.00	2.30	97.30	2.10	101.70	2.40	97.10	2.27	98.70
CAL6	674.00	1.10	101.80	1.80	99.10	3.20	106.10	2.03	102.33
Lorazepam									
CAL1	24.90	2.20	95.80	8.00	98.30	1.10	102.40	3.77	98.83
CAL2	41.30	0.80	110.40	7.50	104.00	13.30	97.20	7.20	103.87
CAL3	104.00	2.90	102.60	2.10	96.90	5.60	98.00	3.53	99.17
CAL4	210.00	2.20	98.60	0.10	100.20	1.60	96.50	1.30	98.43
CAL5	368.00	8.10	93.80	5.40	100.80	6.20	101.50	6.57	98.70
CAL6	780.00	1.50	101.00	0.90	99.90	2.10	104.50	1.50	101.80
Cocaine									
CAL1	16.90	6.00	98.30	1.70	103.10	2.10	98.30	3.27	99.90
CAL2	26.60	2.80	105.20	2.50	94.30	4.10	101.40	3.13	100.30
CAL3	64.20	2.40	97.20	2.60	101.20	0.10	104.90	1.70	101.10
CAL4	128.00	2.30	98.40	1.00	101.30	4.50	96.90	2.60	98.87
CAL5	223.00	3.10	102.90	2.20	101.00	3.80	100.40	3.03	101.43
CAL6	473.00	1.20	98.80	5.90	99.10	5.80	98.10	4.30	98.67
BEG									
CAL1	15.50	10.70	99.40	0.50	102.40	2.70	98.50	4.63	100.10
CAL2	24.80	8.80	103.40	10.50	97.30	0.50	102.70	6.60	101.13
CAL3	61.70	0.10	94.40	3.20	96.40	1.00	99.80	1.43	96.87
CAL4	122.00	1.50	99.10	5.40	101.80	1.40	97.60	2.77	99.50
CAL5	213.00	6.30	104.70	1.30	98.10	3.50	102.90	3.70	101.90
CAL6	455.00	1.40	99.20	5.70	104.00	4.30	98.50	3.80	100.57
Phenobarbital									
CAL1	77.00	11.00	95.50	6.00	95.20	13.40	100.00	10.13	96.90
CAL2	124.00	4.80	105.90	7.00	107.20	14.10	98.60	8.63	103.90
CAL3	305.00	7.00	103.70	11.20	102.00	4.20	104.00	7.47	103.23
CAL4	615.00	2.20	101.00	6.40	102.40	4.20	101.90	4.27	101.77
CAL5	1107.00	3.70	98.80	8.70	93.00	4.60	95.60	5.67	95.80
CAL6	2451.00	3.90	95.10	2.30	100.20	4.00	100.00	3.40	98.43
Morphine									
CAL1	13.50	4.70	97.60	1.60	100.40	0.60	98.90	2.30	98.97
CAL2	21.30	5.80	105.90	2.80	98.40	3.10	101.30	3.90	101.87
CAL3	52.10	1.10	100.00	2.90	102.60	1.40	101.40	1.80	101.33
CAL4	104.00	0.70	98.60	1.90	100.80	0.70	99.40	1.10	99.60
CAL5	181.00	2.20	99.40	3.60	98.40	0.00	100.70	1.93	99.50
CAL6	383.00	0.90	99.50	3.70	99.40	0.40	98.30	1.67	99.07
6-MAM									
CAL1	2.68	9.90	98.10	1.70	102.60	0.70	99.50	4.10	100.07
CAL2	4.31	N/A	108.30	6.20	93.90	8.00	102.30	7.10	101.50
CAL3	11.20	0.80	101.00	9.60	105.00	6.00	95.00	5.47	100.33
CAL4	22.90	7.30	100.70	6.90	100.20	2.50	102.70	5.57	101.20
CAL5	40.80	0.20	102.20	0.20	101.60	1.10	99.40	0.50	101.07
CAL6	90.20	1.40	94.90	3.70	96.80	2.00	101.20	2.37	97.63
Methadone									
CAL1	28.50	5.80	99.00	0.20	101.20	0.80	100.30	2.27	100.17
CAL2	45.50	6.10	104.00	2.20	99.00	0.90	100.40	3.07	101.13
CAL3	112.00	0.60	96.80	2.10	98.20	0.60	99.00	1.10	98.00
CAL4	221.00	3.40	97.10	1.70	98.80	0.60	96.00	1.90	97.30
CAL5	378.00	1.10	100.00	5.40	98.80	3.70	101.50	3.40	100.10
CAL6	773.00	0.40	103.60	4.90	104.00	0.80	102.70	2.03	103.43
EDDP									
CAL1	26.00	4.10	99.40	2.70	100.00	2.80	100.40	3.20	99.93
CAL2	40.80	6.30	102.60	2.80	100.00	2.70	100.30	3.93	100.97
CAL3	99.50	2.00	97.30	1.70	101.70	0.70	98.00	1.47	99.00
CAL4	196.00	1.70	99.20	0.30	97.20	0.60	97.90	0.87	98.10
CAL5	341.00	2.10	100.20	3.90	98.50	2.90	102.30	2.97	100.33
CAL6	716.00	3.60	101.60	2.20	102.70	2.50	101.10	2.77	101.80
Ketamine									
CAL1	26.60	3.90	96.00	6.40	101.90	2.80	99.40	4.37	99.10
CAL2	42.70	7.10	111.30	1.70	97.70	8.00	101.00	5.60	103.33
CAL3	106.00	7.50	98.10	5.80	97.90	5.00	101.10	6.10	99.03
CAL4	216.00	2.30	95.70	3.30	100.40	0.80	96.10	2.13	97.40
CAL5	382.00	0.30	98.40	3.50	99.20	4.00	102.30	2.60	99.97
CAL6	841.00	1.80	102.50	3.00	102.90	3.60	100.10	2.80	101.83

#### Sensitivity

 In order to determine LOD and LOQ values, corresponding to the lowest concentrations of drugs and metabolites, whose signal was distinguishable from background or attributable to an accurate value, dilutions of CAL1 were performed. LOD and LOQ values are reported in Table 2. As expected, LOD values are greatly lower than CAL1. Moreover, LOQ values correspond to CAL1 for MDMA, clonazepam, cocaine, BEG, phenobarbital, 6-MAM, methadone, and EDDP. All analytes’ LOD and LOQ values were also reported in S1_validation.

**Table 2:** LOD (µg/L) and LOQ (µg/L) values of representative substances belonging to the classes of amphetamines, benzodiazepines, barbiturates, opioids, and other

Compound	LOD (µg/L)	LOQ (µg/L)
Amphetamine	2.17	10.85
MDMA	0.40	25.80
Clonazepam	0.40	25.60
Lorazepam	2.07	10.33
Cocaine	0.13	16.90
BEG	1.24	15.50
Phenobarbital	31.00	77.00
Morphine	0.21	1.08
6-MAM	0.22	2.68
Methadone	4.55	28.50
EDDP	2.04	26.00
Ketamine	0.43	10.68

#### Precision and accuracy

Precision and accuracy of the analytical method was evaluated testing 3 levels of QC intra-daily and inter-daily. As shown in Table 3, CV (%) was not > 15% and ACC (%) did not exceed 100 ± 15% of the nominal value.

**Table 3:** Intra-day and inter-day precision, expressed as coefficient of variation (CV (%)), and accuracy, expressed as percentage of accuracy (ACC (%)) of QC

Standard	Nominal concentration (µg/L)	Intra-day						Inter-day	
		I		II		III			
		CV (%)	ACC (%)	CV (%)	ACC (%)	CV (%)	ACC (%)	CV (%)	ACC (%)
Amphetamine									
QCI	53.90	5.70	100.70	3.30	96.90	0.60	99.30	3.20	98.97
QCII	513.00	8.20	103.70	2.10	98.30	0.60	101.40	3.63	101.13
QCIII	157.00	1.20	100.50	0.50	95.50	1.10	99.10	0.93	98.37
MDMA									
QCI	48.10	8.70	102.90	2.80	100.50	2.30	103.30	4.60	102.23
QCII	515.00	9.00	100.80	3.40	95.20	2.20	97.30	4.87	97.77
QCIII	140.00	0.50	102.00	1.00	98.10	1.80	103.90	1.10	101.33
Clonazepam									
QCI	50.00	3.10	106.70	2.70	96.50	3.40	96.10	3.07	99.77
QCII	506.00	7.40	99.40	4.40	94.60	1.90	93.90	4.57	95.97
QCIII	146.00	5.50	101.70	2.50	97.00	1.20	96.40	3.07	98.37
Lorazepam									
Q CI	51.40	9.50	96.90	3.60	95.90	3.80	96.40	5.63	96.40
QCII	577.00	8.90	98.10	2.10	96.50	3.90	97.00	4.97	97.20
QCIII	156.00	5.40	100.70	1.60	96.50	2.50	100.30	3.17	99.17
Cocaine									
QCI	32.70	4.00	104.40	5.80	99.40	2.90	96.30	4.23	100.03
QCII	347.00	5.50	100.50	3.30	98.70	3.10	96.70	3.97	98.63
QCIII	95.10	1.20	107.00	5.00	102.10	4.40	98.60	3.53	102.57
BEG									
QCI	30.80	5.80	102.80	2.80	102.10	4.40	104.10	4.33	103.00
QCII	336.00	7.90	103.60	4.70	94.70	1.70	97.10	4.77	98.47
QCIII	91.40	0.90	99.20	3.40	96.40	2.70	103.10	2.33	99.57
Phenobarbital									
QCI	153.00	7.70	102.60	7.90	91.30	9.70	96.60	8.43	96.83
QCII	1754.00	9.20	100.30	4.10	94.80	3.20	97.60	5.50	97.57
QCIII	462.00	6.50	107.10	7.20	99.50	2.30	101.90	5.33	102.83
Morphine									
QCI	26.50	6.00	102.70	1.70	98.80	2.00	100.00	3.23	100.50
QCII	281.00	8.30	99.20	2.60	95.70	0.70	97.10	3.87	97.33
QCIII	78.20	1.20	101.10	1.50	98.10	1.30	100.30	1.33	99.83
6-MAM									
QCI	5.47	7.30	110.50	5.00	88.10	13.00	101.70	8.43	100.10
QCII	64.10	8.90	99.40	1.50	97.30	5.20	94.10	5.20	96.93
QCIII	16.80	1.60	106.00	9.40	103.10	6.20	95.00	5.73	101.37
Methadone									
QCI	56.40	4.30	102.10	2.30	98.50	0.80	97.60	2.47	99.40
QCII	577.00	6.70	104.00	3.00	98.80	1.40	96.80	3.70	99.87
QCIII	165.00	1.70	99.70	0.90	96.70	1.40	98.10	1.33	98.17
EDDP									
QCI	50.60	5.80	101.80	1.50	97.10	0.30	98.20	2.53	99.03
QCII	526.00	8.80	101.30	2.10	95.60	1.50	95.30	4.13	97.40
QCIII	147.00	0.60	100.50	1.30	97.80	0.50	100.30	0.80	99.53
Ketamine									
QCI	53.10	8.10	105.40	5.30	97.00	5.40	96.00	6.27	99.47
QCII	601.00	4.90	104.50	3.40	105.00	0.80	97.30	3.03	102.27
QCIII	160.00	3.10	105.60	1.30	96.30	3.00	95.00	2.47	98.97

#### Carry-over

 Carry-over was evaluated injecting blank after the CAL with the highest concentration: blank signal resulted to be lower than 20% and 5% of those of analytes at CAL1 and IS, respectively, at the retention times (S1_validation).

#### Matrix effect, recovery, and process efficiency

Matrix effect (ME) (i.e., differences in responses of analytes due to the biological matrix), as well as recovery (RE) (i.e., proportion of analytes extracted from the sample preparation) and process efficiency (PE), phenomenon comprising the above-mentioned effects that deal with the efficiency of the entire process, were evaluated for all 3 levels of QC. We choose to show the results of some substances representative of the main drugs of abuse classes: ME, RE, and PE did not exceed 100 ± 20% (Table 4), similarly to what was observed also for the other analytes tested (S1_validation).

**Table 4:** Matrix effect (ME), recovery (RE), and process efficiency (PE) values referred to representative substances belonging to the classes of amphetamines, benzodiazepines, barbiturates, opioids, and other

Compound	ME (%)			RE (%)			PE (%)		
	QCI	QCII	QCIII	QCI	QCII	QCIII	QCI	QCII	QCIII
Amphetamine	99.52	100.02	95.24	104.50	107.87	107.67	104.00	107.89	102.55
MDMA	99.58	94.54	89.18	102.25	102.64	114.73	101.83	97.03	102.32
Clonazepam	100.52	98.61	87.23	107.99	103.62	117.11	108.55	102.18	102.16
Lorazepam	100.89	89.38	94.32	100.29	114.11	110.28	101.19	101.99	104.02
Cocaine	85.15	97.64	91.48	101.83	106.32	105.90	86.70	103.81	96.88
BEG	92.05	93.71	88.65	114.27	111.08	110.29	105.18	104.10	97.77
Phenobarbital	116.25	94.49	105.35	102.32	91.48	113.40	118.95	86.43	119.47
Morphine	99.84	95.40	93.98	107.29	106.94	108.02	107.12	102.02	101.52
6-MAM	79.52	94.99	96.58	103.24	116.18	104.71	82.09	110.36	101.13
Methadone	98.48	95.08	89.09	118.41	115.13	114.35	116.61	109.46	101.88
EDDP	95.70	96.41	91.69	113.48	109.93	106.26	108.60	105.98	97.43
Ketamine	99.61	90.80	86.10	106.61	115.71	108.04	106.19	105.06	93.03

Unfortunately, testing these parameters, we found out that the opioid buprenorphine and the metabolite of cannabinoids 11-Nor-9-carboxy-Δ9-tetrahydrocannabinol (THC-COOH) presented suppression of the signal due to the matrix (S1_validation).

#### Stability

Stability of analytes in bile diluted 1:100 and stored at -20 °C was evaluated. In particular, after 1 month, all analytes resulted unchanged in concentration and therefore undegraded (data not shown).

Forensic application.

In order to assess the applicability of the proposed LC–MS/MS method, postmortem specimens derived from 13 individuals were analyzed. Raw results from the quantification in these samples were reported in S1_validation. In particular, evaluating the illicit drugs and their metabolites detected, methadone (70%) and cocaine (60%) resulted to be the most prevalent consumed substances, followed by morphine and the benzodiazepines alprazolam, clonazepam, and diazepam (30%). Tramadol and Δ9-tetrahydrocannabinol (23%), zolpidem (15%), and amphetamine, pregabalin, and ketamine (7%) were detected in a lower extent.

Interestingly, norcodeine and 6-MAM, products of metabolism of codeine and morphine, respectively, were not detected in blood. Also, lormetazepam was found only in urine and metabolites after deconjugation. Furthermore, Δ9-tetrahydrocannabinol (THC) was detected only in blood, consistently with the levels of the metabolite THC-COOH detected in urine and bile.

When compounds were quantified in all the matrices tested, bile:blood and bile:urine ratios were determined (Table 5). Interestingly, the concentrations of all the compounds, except for tramadol and cocaethylene, were higher in bile than in blood. Moreover, detection in bile instead of the one in urine could be advantageous for identifying the administration of clonazepam and THC-COOH.

**Table 5:** Mean ± standard deviation (SD) of the concentrations detected in biological matrices, number of forensic cases for each compound detected, bile:blood and bile:urine ratios. NA not available

Compounds	Blood concentrations			Urine concentrations			Bile concentrations			Bile:blood ratio	Bile:urine ratio
	Mean	SD	n. cases	Mean	SD	n. cases	Mean	SD	n. cases		
*Amphetamine*	3.80	NA	1	331.10	NA	1	111.00	NA	1	29.21	0.34
*Alprazolam*	26.30	21.04	4	258.50	270.23	4	380.63	147.74	3	14.47	1.47
*OH-Alprazolam*	1.10	NA	1	329.73	133.39	4	747.48	789.64	4	679.53	2.27
*Clonazepam*	49.05	51.97	2	75.40	83.27	3	741.00	940.89	3	15.11	9.83
*Amino-clonazepam*	3107.33	5781.96	4	1553.60	1684.43	4	4330.74	5699.59	4	1.39	2.79
*Diazepam*	118.20	183.03	4	20.33	25.15	3	127.00	40.63	3	1.07	6.25
*Nor-diazepam*	339.08	563.64	4	161.80	113.98	3	362.90	84.52	4	1.07	2.24
*Lorazepam*	104.93	54.59	3	1785.65	1705.01	4	532.81	507.94	4	5.08	0.30
*Oxazepam*	27.10	NA	1	369.88	431.50	4	343.70	481.82	4	12.68	0.93
*Temazepam*	89.40	NA	1	1036.10	1572.50	4	578.33	666.15	4	6.47	0.56
*Pregabalin*	727.90	NA	1	39,015.00	NA	1	1094.00	NA	1	1.50	0.03
*Cocaine*	612.58	1149.80	5	3040.27	3187.98	7	1882.92	3641.48	6	3.07	0.62
*Cocaethylene*	90.40	NA	1	NA	NA	NA	27.13	NA	1	0.30	NA
*Norcocaine*	40.90	62.03	3	141.20	102.71	5	150.69	179.92	6	3.68	1.07
*BEG*	1571.66	2033.83	7	11,013.35	12,115.47	8	3205.54	4528.54	8	2.04	0.29
*THC-COOH*	65.50	35.95	3	491.00	179.66	3	51,929.04	60,714.19	5	792.81	105.76
*Codeine*	5.80	3.68	2	711.95	100.48	2	19.28	18.00	2	3.32	0.03
*Morphine*	25.60	13.45	4	25,374.50	34,312.11	4	6998.40	6065.81	4	273.38	0.28
*Tramadol*	1655.80	2814.00	3	47,769.83	78,593.50	3	485.59	734.17	4	0.29	0.01
*O-DM-Tramadol*	191.50	298.51	3	8275.73	11,117.55	3	877.00	1099.01	3	4.58	0.11
*Methadone*	1071.25	1304.87	8	4623.99	2864.03	8	6054.48	3700.87	8	5.65	1.31
*EDDP*	240.86	317.44	8	6124.77	7096.98	9	23,424.46	20,963.69	9	97.25	3.82
*Zolpidem*	23.80	NA	1	86.70	106.21	2	76.93	82.13	2	3.23	0.89
*Ketamine*	4.40	NA	1	35.60	NA	1	31.30	NA	1	7.11	0.88

## Discussion

The proposed LC–MS/MS method allowed confirmation of the identity, as well as the quantification, of a substantial number of illicit drugs and their metabolites in bile in postmortem cases of SD occurring both for overdose in adults and for intoxication in children.

Drugs that are lipophilic and with high molecular weight are more prone to undergo hepatic metabolism and excretion via biliary route (Ghibellini et al. 2006). Biliary elimination may impact on the pharmacological effect and toxicity of drugs, even because of reabsorption along the gastrointestinal tract, but so far there is limited information regarding the illicit drugs kinetics because of the difficulties to obtain bile samples from healthy subject (Ghibellini et al. 2006). Several illicit drugs, and in particular the metabolites produced after hepatic metabolism, were found in bile in higher amount: for instance, benzodiazepines were detected in their hydroxylated form, as well as natural and synthetic opioids in their conjugated form (Al-Asmari 2020; Bertagni et al. 1972; Kuwayama et al. 2008; Misra et al. 1977; Tominaga et al. 2016).

Measuring drugs concentration in bile is of forensic interest because of the need to test at least 2 matrices since sometimes the traditional ones are not available and the toxicology laboratory could meet technical difficulties due to putrefaction mechanisms in the biological materials collected (Ojanperä et al. 2012). Furthermore, there is evidence about the extended detection capability in bile: the concentration of several drugs are up to 520 fold times higher than the ones found in blood (Ferner and Aronson 2018). Other alternative matrices, such as vitreous humor and cerebrospinal fluid, could be employed but they need the addition of stabilizers (Bévalot et al. 2016a) or are characterized by low concentration of drugs (Tominaga et al. 2015).

To the authors’ knowledge, this is the first time a validated LC–MS/MS was used to screen such a large quantity of drugs of abuse: previous works were focused on the quantification of only one analyte or class of analytes using this technique (Al-Asmari 2019, 2020; Zuccarello et al. 2023). Using the same analytical method for several drugs in different matrices gains in value because it allows a more accurate comparison between the classes of drugs from a pharmacokinetic point of view and between the different specimens available for the analysis (Ferner and Aronson 2018; Vanbinst et al. 2002). However, the present analytical method did not focus on antidepressant drugs, as other studies did before (Zuccarello et al. 2023). Furthermore, other techniques, such as GC–MS, were typically employed for achieving the aim of the present paper (Tominaga et al. 2016).

In the proposed method, undiluted bile resulted unmanageable for analysis because of its influence on both chromatography and ionization; indeed the biological material presented a very low pH, probably due to putrefaction mechanisms (Donaldson and Lamont 2013). Instead, bile diluted 1:100 resulted to be the best material to work with since it allowed quantification of the analytes without having a significant matrix effect. Such a large dilution allowed to use a small amount of matrix, contrary to what was observed for other analytical methods (Tominaga et al. 2016).

Sample preparation of the present method is fast, simple and consists mainly in enzymatic deconjugation and protein precipitation through organic solvents. Since several drugs undergo conjugation through hepatic metabolism, enzymatic hydrolysis needs to take place to detect the original molecule; classic examples are opioids that are excreted for a large extent as glucuronide products (Concheiro et al. 2018). Furthermore, this sample preparation did not use solid-phase extraction, a time-consuming further step usually needed for purifying bile, as other previous works did (Bévalot et al. 2016b; Tominaga et al. 2016).

Validation of the analytical method according to the more recent ICH guidelines was also performed to assess an appropriate selectivity, specificity, sensibility, linearity, accuracy, precision, matrix effect, and recovery and applicability to autoptic samples.

Comparing the validation parameters with the ones of previously reported analytical methods, LOD and LOQ values referred to our LC–MS/MS method are greatly lower suggesting that we applied a novel and more sensitive MS/MS equipment in our procedure compared to the former ones (Launiainen and Ojanperä 2014; Vanbinst et al. 2002). The benzodiazepine clonazepam is an exception; in particular, Launiainen and colleagues developed a liquid chromatography/time-of-flight mass spectrometry (LC-TOF–MS) method with a LOQ value of 10 µg/L for clonazepam in order to investigate femoral blood concentration of drugs (Launiainen and Ojanperä 2014). Furthermore, Vanbinst et al. considered in their method a larger range of linearity, allowing an accurate analysis also at very high concentrations (Vanbinst et al. 2002).

Noteworthy, the already reported postmortem drugs concentration in bile resulted to be higher than our LOD values allowing identification and, in most cases, also the quantification of the illicit drugs and their metabolites found in this matrix (Launiainen and Ojanperä 2014; Tominaga et al. 2016).

Carry-over, defined as the contribution of the response of analytes and IS in the subsequent runs, is a big issue in quantitative analysis; therefore, several regulatory bodies of requirements for method validation, such as ICH and Food and Drug Administration (FDA), fixed specific acceptance criterion (Jogpethe et al. 2022). The present method satisfies this criterion. However, especially in case of high exposed samples, complete elimination of carry-over effect is not possible and there is the need to evaluate if it affects the accuracy and precision of the analytical method (Jogpethe et al. 2022).

Interestingly, matrix effect has never been taken into consideration before. Testing matrix effect, we found that the present method was not ideal for the opioid buprenorphine and the metabolite of cannabinoids 11-Nor-9-carboxy-Δ9-tetrahydrocannabinol because of the suppression due to the matrix.

Tominaga investigated the recovery and reported values from 60 to 70% for phenobarbital to > 95% for codeine (Tominaga et al. 2016). Instead, 7-amino nitrazepam resulted in the molecule with a lower recovery (79%) in our study.

The present work has the purpose also to test the applicability of the LC–MS/MS method on real samples derived from postmortem specimens and to compare the concentrations of analytes in bile with the ones of the traditional matrices (blood and urine). Beyond urine, peripheral blood was used for the comparison because it is less likely be subject to postmortem drug redistribution, referred as changes in drugs concentration due to organ injury and subsequent release of its content in blood (Yarema and Becker 2005). Also, it is important to notice that blood is representative of the potentially lethal concentration at the time of the death, instead bile and urine may demonstrate past drug exposure that could also be indicative (Bierly and Labay 2018). Indeed, as expected, products of hepatic metabolism, such as norcodeine and 6-MAM (Smith 2009), were detected in bile and urine, but not in blood.

Several works investigated bile:blood ratio in order to find a fixed relationship between the levels in the matrices (Ferner and Aronson 2018). On the basis of these evidences, we calculated bile:blood and bile:urine ratios, since bile contains drugs already metabolized. Even if our results did not differ greatly from the ones reported by Ferner et al., there is no fixed relationship, even taking into account the classes of illicit drugs (Ferner and Aronson 2018).

Even if to date there is no fixed relationship between the several matrices compared, the inter-matrix ratio resulted to be in line with the toxicokinetic of the illicit drugs tested and with previous works. Generally, comparing urine and bile concentrations, there is no wide difference since they, unlike blood, accumulate drugs after hepatic metabolism. The only metabolite that is greatly higher in bile than in urine is THC-COOH, probably due to the postmortem distribution of blood to bodily fluids and tissues and the accumulation in bile following multiple doses, as another paper has already reported (Zughaibi et al. 2023). Furthermore, the bile:blood ratio gave interesting information. For instance, hydroxylation is an important metabolic step for elimination of benzodiazepines, particularly for alprazolam (Ait-Daoud et al. 2018). After abuse of natural and synthetic opioids, their derivatives, such as 6-MAM and EDDP, were found only or in greatly higher amount in bile, as expected (Al-Asmari 2020; Baselt and Casarett 1972). Furthermore, only the concentrations of tramadol and cocaethylene were lower in bile than in blood. In the former case, this could be due to the metabolism of tramadol in O-DM-tramadol, instead the latter to the metabolite half-life, longer than cocaine and its other metabolites (Andrews 1997; Gong et al. 2014).

In conclusion, our results generally confirmed higher concentrations in bile than in blood suggesting a potential role of bile in helping pathologists to identify the cause of death. Based on our data, quantification of drugs of abuse in bile resulted convenient in most cases, except for tramadol and cocaethylene.

### Supplementary Information

Below is the link to the electronic supplementary material.

Supplementary file1 (XLSX 2997 kb)

## Data Availability

The datasets from the LC–MS/MS analyses are available from the corresponding author on reasonable request.
